# Sound can enhance the analgesic effect of virtual reality

**DOI:** 10.1098/rsos.150567

**Published:** 2016-03-30

**Authors:** Sarah Johnson, Matthew Coxon

**Affiliations:** Faculty of Health and Life Sciences, York St John University, Lord Mayors Walk, York YO31 7EX, UK

**Keywords:** pain tolerance, pain perception, virtual reality, head-mounted display

## Abstract

Virtual reality (VR) technology may serve as an effective non-pharmacological analgesic to aid pain management. During VR distraction, the individual is immersed in a game presented through a head-mounted display (HMD). The technological level of the HMD can vary, as can the use of different input devices and the inclusion of sound. While more technologically advanced designs may lead to more effective pain management the specific roles of individual components within such systems are not yet fully understood. Here, the role of supplementary auditory information was explored owing to its particular ecological relevance. Healthy adult participants took part in a series of cold-pressor trials submerging their hand in cold water for as long as possible. Individual pain tolerances were measured according to the time (in seconds) before the participant withdrew their hand. The concurrent use of a VR game and the inclusion of sound was varied systematically within participants. In keeping with previous literature, the use of a VR game increased pain tolerance across conditions. Highest pain tolerance was recorded when participants were simultaneously exposed to both the VR game and supplementary sound. The simultaneous inclusion of sound may therefore play an important role when designing VR to manage pain.

## 1. Background

Virtual reality (VR) technology can provide an effective non-pharmacological means to reduce acute and traumatic injury pain [1]. This technique typically requires participants to experience a virtual digital environment through a combination of computer peripherals. These may include a head-mounted display (HMD) with head-tracking, headphones that provide sound and/or noise reduction and handheld input devices (such as game controllers). Participants make use of the technology to take part in an immersive VR experience, usually an interactive game, while experiencing some form of pain. Such system designs have been found to be effective in minimizing the perception of the pain in a range of clinical contexts including: treatment and care of burn victims [1,2]; paediatric cancer treatment [3,4] and dental pain [5,6]. These systems have also been effective in laboratory tests with healthy adults [7–10]. However, the relative effectiveness of the design depends upon the technological level of the system used [11].

Hoffman *et al*. [11] found that perceived pain was reduced to a greater extent in a ‘high technology’ group compared with a ‘low technology’ group. The system design differed in terms of: the field of view of the HMD; graphical resolution; game interactivity; head-tracking and inclusion of sound. Efforts to determine the influence of each individual component are ongoing. For example, Hoffman *et al*. [12] found that an HMD with a larger field of view was more effective than one with a smaller field of view; Dahlquist *et al*. [7] and Wender *et al*. [13] found that an interactive game was more effective than a non-interactive game; Dahlquist *et al*. [9] found that changing the avatar point of view made no difference for their sample, whereas Law *et al*. [14] have isolated the effect as an attentional one discrete from the kinaesthetic aspects of controller use. An important aspect of the design which has yet to be directly explored is the importance of including sound within such a system.

It is possible that sound may have an additive role (enhancing the overall analgesic effect for the participant), that sound may be unnecessary (adding nothing to the analgesic effect) or that sound on its own may be sufficient (providing an equivalent analgesic effect). On a theoretical level, it has been suggested that the analgesic effect of VR may be due to competing demands for attentional resources [11,15] in line with theoretical models of pain perception in which pain must be attended to in order to be perceived [16]. This deleterious effect has been framed according to the multiple resource theory of Wickens [17]. Within this theory, it is proposed that attentional resources may be distributed across different sensory systems such that attentional demands on one sensory system may not necessarily deplete the resources available for other sensory systems. It has been argued that highly engaging and interactive VR designs should therefore provide greater distraction as they will deplete the attentional resources across multiple sensory systems [7–9,14]. With the individual component of sound, it is therefore viable that the inclusion of sound alongside a VR experience will demand additional attentional resources that are otherwise not engaged, that this competition will limit the overall attentional resources available for pain perception, and in turn enhance the analgesic effect. This assumption has yet to be tested.

This assumption is particularly important on an ecological level. The ultimate aim of such research is to be able to sensibly advise practitioners on the most effective designs for their VR systems. In practice, many practitioners are unlikely to have the resources to be able to make use of large-scale and highly immersive VR systems and are likely to be limited to affordable commercially available systems. Recent developments have meant that the use of HMDs is becoming increasingly more affordable and relatively low-cost headsets are soon to reach commercial markets. If an individual decides to make use of this new low-cost technology, a simple decision a practitioner can make is whether to include sound as part of the distraction technique through the addition of a pair of headphones. On a theoretical level, we would assume that this should make a positive difference but as yet there is no direct empirical evidence to support such a claim. Whether sound does make a difference is therefore a question of ecological relevance.

Here, participants took part in a series of cold-pressor trials to measure pain tolerance. The inclusion of sound was systematically manipulated alongside playing a VR game with an HMD such that participants were exposed to: (i) no sound and no HMD (baseline); (ii) sound but no HMD; (iii) HMD but no sound; and (iv) a combination of both the sound and the HMD. In line with theoretical accounts of pain perception [16], it was expected that sound would have an additive effect, increasing pain tolerance when included with the VR game. It was also expected that it would have an analgesic effect in its own right separate from any influence of the VR game.

## 2. Methods

Thirty-two healthy adults were recruited from York St John University campus (23 females, nine males) with a mean age of 20.28 years (s.d. = 2.00). Both written and verbal consent was obtained prior to taking part in the study using procedures approved by the relevant research ethics committee at York St John University. Participants were excluded from participating if they had a known condition or health issue that may have interacted with the use of an HMD (e.g. existing feelings of nausea).

### 2.1. Design

A within-participants design was used with each participant acting as their own control group. Participants completed four cold-pressor trials under four different conditions: no HMD and no sound (baseline); sound only; HMD only, and both HMD and sound. To control for order effects, all participants provided the baseline measure first before completing the remaining three conditions in a randomized order. Pain tolerance was measured as the total number of seconds the participant kept their non-dominant hand submerged in the ice water during each condition.

### 2.2. Equipment

**Figure 1.**
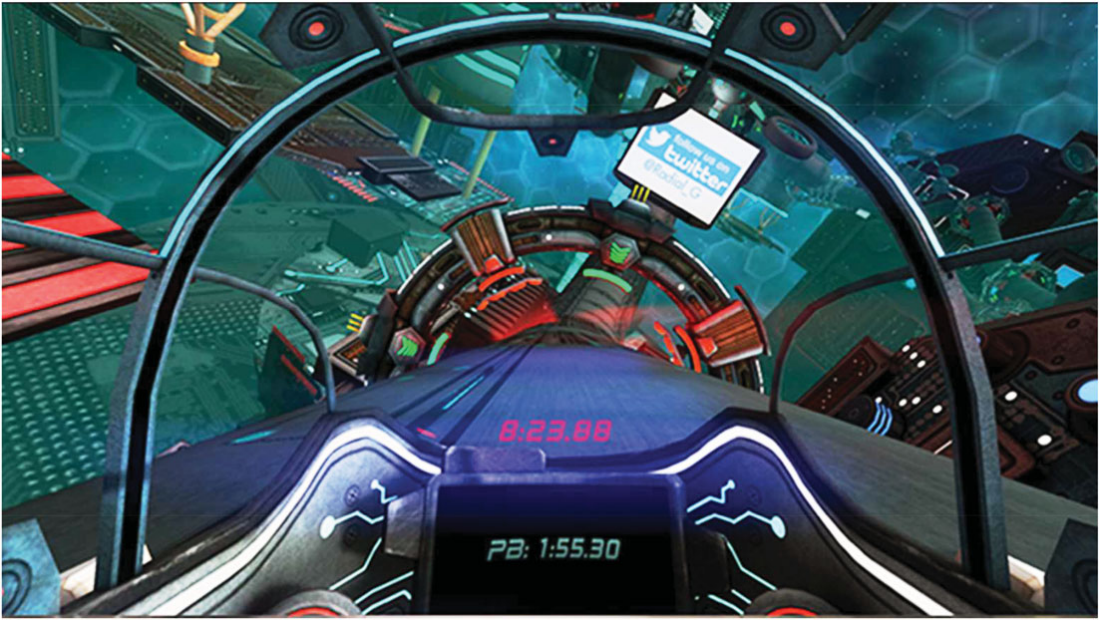
An example of the participant's view in *Radial-G*.

A demonstration version of *Radial-G* (Tammeka Games) was chosen as the VR game for this study. *Radial-G* is an interactive futuristic racing game played from a first person perspective; a player is seated in a space vehicle and races around a track alone (figure 1). Players cannot die in the game, and the game does not end until it is manually stopped. The speed and direction of the virtual vehicle can be controlled using the arrow buttons on a keyboard.

The HMD was an Oculus Rift DK2 with a resolution of 960 × 1080 pixels per eye, a 100° field of view and a 75 Hz refresh rate. The HMD used low-persistence organic light-emitting diodes, and provided both head-tracking and positional tracking. It is worth noting that the Oculus Rift is not a medical device and has not been approved by the US Food and Drug Administration. The HMD was connected to an Alienware 17 laptop with a fourth-gen Intel(R) Core(TM) i7-4710MQ processor and NVIDIA(R) GeForce(R) GTX880M graphics card. Noise cancelling headphones were manufactured by Sennheiser (PXC, 250-II) and were used to play the game-corresponding music. The game music was the same for all conditions where it was played. The noise cancelling function was turned on whenever the headphones were worn.

A Fastime Zero 1 stopwatch was used, and both hand temperature and water temperature were measured using a biofeedback probe manufactured by Electronic Temperature Instruments Ltd., with a detectable temperature range between −19.9 and 69.9°C. The cold-pressor equipment consisted of a container filled with cold water maintained at 1°C using cooler blocks and ice. The water was maintained around 1°C to lower the possibility of ceiling effects but nevertheless provide an appropriate pain stimulus [8]. The temperature was allowed to fluctuate between 1°C above or below the target temperature. A bucket of warm water was used to reheat the participant's hand once they had removed it from the cold water. It is worth noting that additional measures of ‘presence’ were completed for the two conditions making use of an HMD for exploratory reasons. The results of these are not reported here as they apply only to two conditions, and the information is tangential to the specific aims of the research.

### 2.3. Procedure

Participants were seated with their non-dominant hand next to the cold-pressor apparatus and in front of the laptop, HMD and headphones. Before each condition, the participant's hand temperature was attained using the biofeedback probe. After hand temperature was attained, the probe was placed in the cold-pressor to monitor the water temperature. Participants wore noise cancelling headphones across all conditions.

A baseline measure of pain tolerance was taken first. Participants were asked to submerge their non-dominant hand in the cold water and to hold it there for as long as possible. Time was recorded from the point at which the participants hand was submerged up to the wrist and stopped when the hand was removed. Immediately after the participant removed their hand, it was placed in warm water. The temperature of the hand was then measured to ensure it had returned to the baseline temperature. Single cold-pressor trials were then completed for the remaining three conditions in a randomized order. The temperature of the warm water bath was monitored between trials and was constant.

In the sound only condition game, corresponding music was played through the headphones during the trial, and no HMD was worn. The visual input in this condition was therefore identical to that in the baseline. In the HMD only condition, the game was played during the trial wearing the HMD and controlling the game using their dominant hand and the arrow keys on the laptop. In the sound and HMD condition, both game-corresponding music and the game were played. Participants were given the opportunity to practise playing the game using the HMD for 30 s before they began the relevant trials. In line with previous research [7–9], a 4 min limit was imposed on participants submerging their hand in the cold water for safety reasons. If participants had not removed their hand after 4 min, they were instructed to do so.

## 3. Results

Times from five participants were removed from the analysis as the participants reached the 4 min safety limit on one or more of the trials (three males and two females). Pain tolerance times for the remaining participants (*n* = 27) were compared across the four conditions using a one-way ANOVA. A log_10_ transformation of the data was used as the distributions of the raw scores were not normal (descriptive statistics is provided in table 1). Skew and kurtosis levels between −1 and +1 were considered as tolerable limits. A significant main effect of condition was found (*F*_3,78_ = 54.53, *p *< 0.05, *η*^2^ = 0.68, *post hoc* power = 1.00) indicating that differences in pain tolerance between the conditions were statistically significant. *Post hoc* pairwise comparisons with a Bonferroni adjustment indicated that mean pain tolerances across all four conditions were statistically different from one another (*p *< 0.05). Scores were lowest in the baseline condition followed by the sound only condition, and scores were higher in the HMD only condition and highest in the sound and HMD condition.

**Table 1.** Descriptive statistics for the pain tolerance scores.

	untransformed times (in seconds)				transformed times (in seconds)			
condition	mean	s.d.	skew	kurtosis	mean	s.d.	skew	kurtosis
baseline	30.14	31.67	2.89	9.28	1.34	0.32	0.84	0.69
sound only	40.30	45.64	2.54	6.07	1.44	0.35	0.81	0.92
HMD only	55.74	51.02	2.14	3.68	1.63	0.30	0.81	0.63
HMD + sound	79.33	66.36	1.28	0.27	1.77	0.34	0.37	−0.69

## 4. Discussion and conclusion

In this study, we compared the relative effectiveness of playing a VR game, listening to sound and the combination of the two on pain tolerance in cold-pressor trials. Participants demonstrated the predicted behaviour with exposure to both a VR game and supplementary sound increasing pain tolerance to a greater extent than the VR game in isolation or the sound in isolation. The results of the study are consistent with the suggestion that the analgesic effect of VR is due to competition for attentional resources [11,15]. The inclusion of sound alongside the VR game is likely to have been more attentionally demanding than both aspects in isolation, thereby leading to less attentional resources that could be allocated to the pain stimulus. This is consistent with the finding that sound on its own also increased pain tolerance, but not to the same extent.

While previous research supports the broad notion that the higher the technological level of the VR system the more effective it will be, this provides limited guidance on the necessary components of such a system. As noted previously, evidence has indicated that the field of view in the HMD [12], and the interactive nature of the game [7,13] may be important. The results reported here have added to this literature through providing the first direct test of whether sound enhances the analgesic effect of VR distraction techniques. In using VR to increase pain tolerance, it is therefore worthwhile including sound within the design of the system wherever possible.

It is worth noting that aspects of the study may limit these conclusions. The participants sampled were predominantly female, and the number of male participants is relatively low. An inspection of the data provides no suggestion that the effects described are more apparent for women rather than men. However, it is worth noting that further research may be needed to guarantee that these specific effects are not more powerful for females than males. On a practical level, it is also worth noting that the apparatus used for the cold-pressor did not include a circulator. The temperature of the water was monitored to ensure that it was at a constant temperature, but the potential for fluctuations in water temperature within the apparatus still remained. This should be borne in mind as minor changes in temperature can influence pain tolerance [18], so these results should ideally be confirmed with the use of a circulator also. Finally, the order of the experimental trials was randomized within participants to control for potential order effects and habituation. The specific and predictable pattern of results despite this randomization would indicate that these aspects were sufficiently controlled for. However, it is important to note that the baseline measure was always taken first. While this is in line with comparable work within the field [7–9], systematic counterbalancing that is inclusive of the baseline may be desirable in future work. This would allow for greater confidence that differences from the baseline measure were not due to other contributory factors related to the order of trials (e.g. habituation, anticipatory anxiety).

On a more conceptual level, future research should also focus upon the nature of the sound used within the design. For example, it would be useful to determine which categories of sound provide the most effective form of distraction from pain. Here, game-relevant music was played, but sounds inconsistent with the game, or distinctive sounds, may instead demand more attentional resources. It would be useful to determine if the sense of immersion provided by game-consistent sound is more or less important than the potential of distinctive sounds to capture attention. It will also be important to determine the limits of such an effect, and the potential contributions of realistic haptic and olfactory stimulation. However, at the moment, a researcher or practitioner may find that their choices are more limited to the use of an HMD and/or headphones when purchasing commercially available equipment. To this end, this study clearly demonstrates the additional benefit of including sound within such systems in addition to the stimulation provided through the HMD.

## Data accessibility

The dataset supporting this article has been made available on the York St John University institutional repository (http://ray.yorksj.ac.uk/960/).
